# Expression and clinical significance of Numb and Notch-1 proteins between tissue of colon cancer and regional lymph node metastases

**DOI:** 10.3389/fonc.2024.1467517

**Published:** 2024-12-03

**Authors:** Jingyou Ma, Jinpeng Zhen, Ningbao Yang, Changjuan Meng, Yanjun Lian

**Affiliations:** The Gastrointestinal Surgery of Xingtai Central Hospital, Xingtai, Hebei, China

**Keywords:** colon cancer tissue, metastatic lymph node tissue, tumor adjacent tissue, Notch-1 protein, Numb protein

## Abstract

**Objective:**

To investigate the expression and clinical significance of Notch-1 and Numb protein in colon cancer tissues and regional lymph node metastases.

**Methods:**

Immunohistochemical method was used to detect the expression of Notch-1 protein and Numb protein in 110 cases of colon cancer tissues, along with tumor adjacent tissues and 56 cases of MLN tissues, and to analyze its role in colon cancer and MLN tissue.

**Results:**

Comparing colon cancer tissue or lymph node metastases with tumor adjacent tissue, the positive expression rate of Numb was significantly decreased, while the positive expression of Notch-1 was significantly increased in colon cancer tissue or lymph node metastases (both *p*<0.05). The expression of Notch-1 and Numb was correlated with the lymph node metastasis, TNM stage, and degree of differentiation (*p*<0.05). The expression between Numb and Notch-1 showed negative correlation in colon cancer tissues (r=−0.261, *p*<0.05). There was no relationship between the expression of Numb and Notch-1 protein in colon cancer and metastatic lymph node tissue (*p*>0.05).

**Conclusion:**

Numb expression is decreased and Notch-1 expression is increased in colon cancer tissue and metastatic lymph node tissue, suggesting that the interaction between the two proteins may play a promote role in the development, invasion, and metastasis of colon cancer. There was no relationship between the expression of Numb and Notch-1 protein in colon cancer and metastatic lymph node tissue, suggesting that there is no obvious enhancement of the cancer cells; in the process of lymph node metastasis, the degree of malignant biological behavior remains relatively stable.

## Introduction

In recent years, the incidence of colon cancer is increasing gradually, and colon cancer has heterogeneity, which requires individualized treatment plan, so personalized treatment has become a new trend in the field of cancer treatment. The key of personalized therapy lies in the high selectivity of tumor targets, and the selection of targeted drugs with good effect and light side effects has become a research hotspot. Molecular targeted therapy can effectively improve the prognosis of cancer patients, improve the survival rate of patients, and become another new method in addition to surgical resection, radiotherapy, and chemotherapy, providing a new treatment for colon cancer patients. Notch-1 protein is involved in cell proliferation, differentiation, apoptosis, etc. (1, 2). The abnormal transmission of Notch-1 signaling pathway may be related to the occurrence of tumors (3). Numb is known as the fate determinant of cell differentiation and can influence cell differentiation in a variety of ways. The role of Numb in the Notch signaling pathway has become the focus of current research (4, 5). It has been suggested that Numb may inhibit the invasion and metastasis of melanoma by regulating NOTCH-CCNE axis, and upregulation Numb inhibitors may play a role in the treatment of melanoma (6). By detecting the expression of Notch-1 protein and Numb protein in colon cancer and MLN, this study provides a possible theoretical basis for the occurrence, development, and metastasis of colon cancer. At the same time, Notch-1 and Numb are targeted to provide a possible molecular basis for the targeted therapy and prognosis of colon cancer.

## Data and methods

### General information

A total of 110 carcinoma tissues and adjacent carcinoma tissues of patients with colon cancer who had surgery in the gastrointestinal surgery of Xingtai Central Hospital in China during October 2019–October 2022 were selected. A total of 56 regional lymph node metastases tissues were selected as samples among the above cases. Of the 110 cases of colon cancer patients, 57 cases were men, 53 cases were women, ages were 41–83 years old, with an average age of 67 years old, and 44 cases were in stage I+II and 66 cases were in stage III+IV. There were 68 cases with tumor diameter ≤5 cm and 42 cases with tumor diameter >5 cm. None of the selected cases received neoadjuvant therapy before surgery and had no intestinal obstruction before surgery. All specimens were treated with radical resection of colon cancer and were pathologically diagnosed as colonic adenocarcinoma.

### Reagent

Numb polyclonal antibody and Notch-1 polyclonal antibody are bought from Proteintech Group, Inc. (Wuhan, Hubei, China).; the kit and citric acid antigen repair reagents were bought from Beijing Zhong Shan -Golden Bridge Biological Technology CO.,LTD (Beijing, China).

### Method

Using the immunohistochemical SP method, the specimens were made of biopsies that were 4 μm, fully hydrated after conventional xylene dewaxing, and repaired by citric acid antigen. Peroxidase is blocked by 3% hydrogen peroxide in each biopsy and incubated for 10 min at room temperature, and the serum was removed; Numb or Notch-1 primary polyclonal antibody is added to each biopsy as a fight and incubated for 90 min at room temperature. Each biopsy was incubated for 30 min at room temperature with the second antibody, added by streptomyces avidin peroxidase reagents, and colored in DAB microscope.

### The result judgment standard

Numb is mainly expressed in the cytoplasm and cell membrane; the yellow particles mean positive cells. Notch-l protein is mainly expressed in the cytoplasm and the nucleus in tan particles; the color intensity of positive cell and the number of positive cells were judged based on the integral semi-quantitative method. Colorless, pale yellow, tan, and brown were marked by 0, 1, 2, and 3, respectively, according to positive staining intensity. Each biopsy was marked by the number of positive cells (0, 1, 2, 3, and 4 points mean <10%, 10%–10%, 26%–50%, 51%–70%, and >70%, respectively). Comprehensive score is equal to the product of two grades, 0 means negative, 1–4 means weakly positive, 5–8 means moderate positive, and >8 means strong positive. Negative and weakly positive scores are viewed as a negative expression; moderate positive and strong positive are viewed as positive expression.

### Statistical processing

The SPSS 21.0 software was used for data analysis, where the significance of count data was compared by χ^2^ test, and the expression correlation of Numb and Notch-1 proteins was analyzed by Spearman rank correlation. *p*-value <0.05 was considered to be statistically significant.

## Results

### The Numb and Notch-1 expression in different tissues

**Figure 1:**
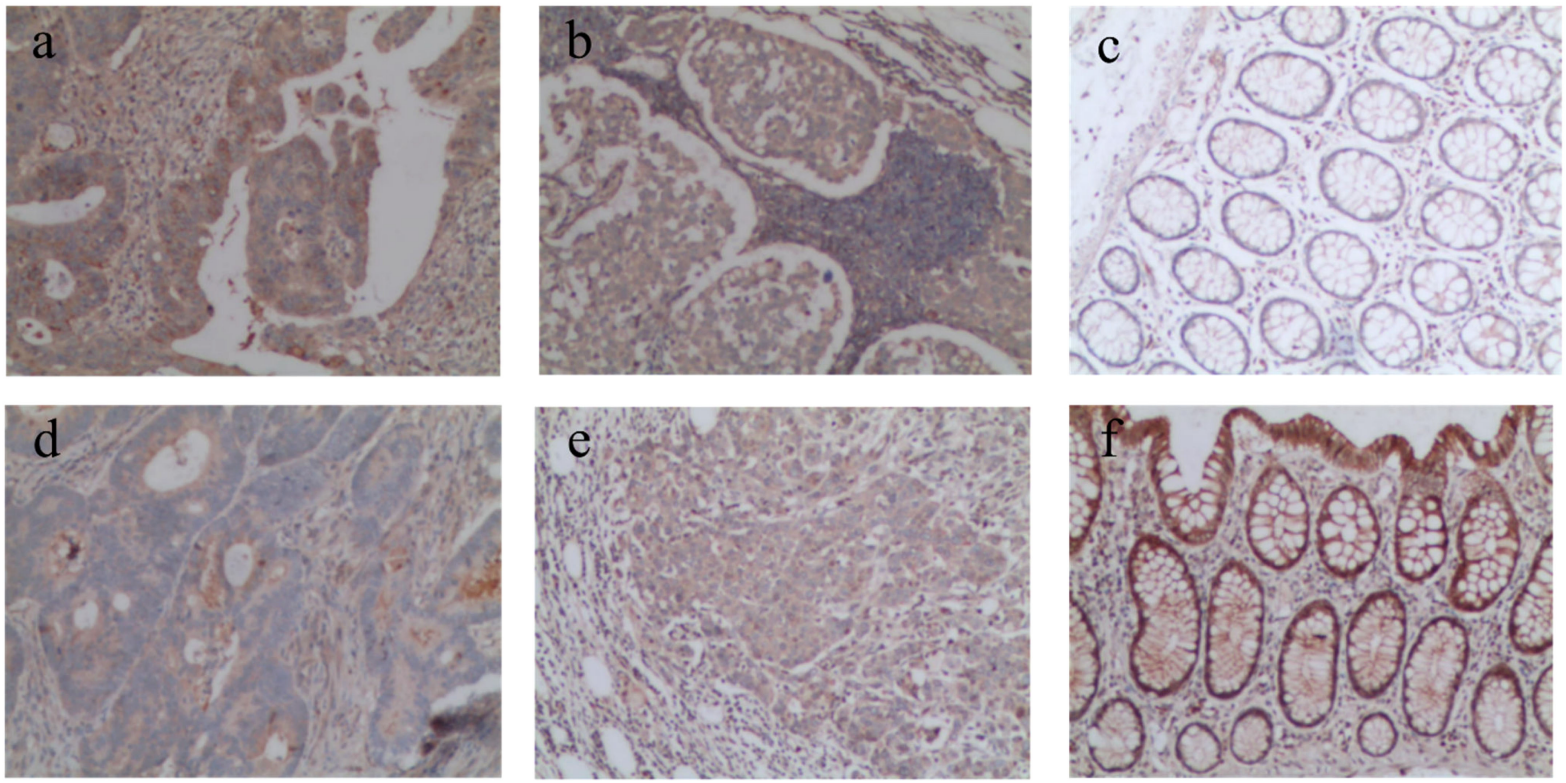
Immunohistochemical detection of Notch-1 protein and Numb protein expression in different tissues (SP × 100). **(A)** Notch-1 is highly expressed in cancer tissues; **(B)** Notch-1 is highly expressed in lymph node metastatic tissues; **(C)** Notch-1 is lowly expressed in tumor adjacent tissues; **(D)** Numb is lowly expressed in cancer tissues; **(E)** Numb is lowly expressed in lymph node metastatic tissues; **(F)** Numb is highly expressed in tumor adjacent tissues.

As shown in **Table 1**, in colon cancer and metastasis lymph node tissues, the positive expression rate of Numb was significantly lower than those in the adjacent normal tissue; the positive expression rate of Notch-1 was significantly higher than that in the adjacent normal tissue (**Figure 1**). The difference was statistically significant (*p* < 0.05). The positive expression rate of Numb or Notch-1 has no statistically significant difference between cancer and metastasis lymph node tissues (*p* > 0.05).

**Table 1:** The Numb and Notch-1 expression in different tissues.

group	*n*	Notch-1		Numb	
		positive	positive rate (%)	positive	positive rate (%)
Cancer tissues	110	71	64.55^①^	34	30.91^④^
Carcinoma Adjacent normal tissue	110	26	23.64^②^	69	62.73^⑤^
Metastatic lymph node tissue	56	39	69.64^③^	14	25.00^⑥^

comparison of each two in different organizations.

①and②: χ^2^=37.340 *p*= 0.000 ①and③: χ^2^=0.431 *p*= 0.511.

②and③: χ^2^=32.968 *p*=0.000: ④and⑤: χ^2^=22.363 *p*=0.000.

④and⑥: χ^2^=0.630 *p*=0.427: ⑤and⑥: χ^2^=21.127 *p*=0.000.

### Clinicopathological factors according to the expression of Numb and Notch-1 protein in 110 patients with colon cancer

As shown in **Table 2**, the expression of Numb was significantly associated in colon cancer with the lymph node metastasis, TNM stage, and degree of differentiation (*p* < 0.05), not with age, gender, serous membrane invasion, and tumor size (*p* > 0.05). The expression of Notch-1 was significantly associated in colon cancer with the lymph node metastasis, TNM stage, and degree of differentiation (*p* < 0.05), not with age, gender, serous membrane invasion,and tumor size (p > 0.05).

**Table 2:** Clinicopathological factors according to the expression of Numb and Notch-1 protein in 110 patients with colon cancer tissues.

Characteristics	*n*	Notch-1			Numb		
		positive	positive rate(%)	*p*	positive	Positive rate(%)	*p*
Age (years)							
≥65岁	87	52	59.77	0.212	26	29.89	0.651
<65岁	23	17	73.91		8	34.78	
Gender							
Male	57	39	68.42	0.378	16	28.07	0.504
Female	53	32	60.38		18	33.96	
Tumor size (cm)							
≤5 cm	68	40	58.82	0.110	24	35.29	0.205
>5 cm	42	31	73.81		10	23.81	
Serous membrane invasion							
Positive	67	46	68.66	0.261	17	25.37	0.117
Negative	43	25	58.14		17	39.53	
Lymph node metastasis							
Positive	66	52	78.79	<0.001	13	19.70	0.002
Negative	44	19	43.18		21	47.73	
TNM stage							
I–II	44	48	72.73	0.028	15	22.73	0.023
III–IV	66	23	52.27		19	43.18	
Differentiation							
Medium–high	61	46	75.41	0.008	12	19.67	0.004
Poor	49	25	51.02		22	44.90	

## Discussion

After the Notch ligand binds to corresponding receptors in the Notch signaling pathway, it causes the division and release of the Notch Intracellular Domain (NICD), acts on downstream target genes, and regulates cell development, proliferation, differentiation, and other activities (7, 8). Notch-1 signaling pathway is related to the occurrence, development, invasion, and metastasis of tumors. However, there are many unexplained factors that affect the Notch signaling pathway. Notch-1, as one of the important members of the Notch family, has gradually become a new trend in the study of colon cancer (9, 10). The most important feature of Numb is the regulation of cell differentiation through asymmetric allocation during mitosis and is therefore known as a determinant of cell fate, and these properties have important implications for the role of Numb in cell physiological development and various diseases (11, 12). Numb and Notch are antagonistic proteins in many literatures (13, 14). Studies have shown that in colorectal cancer, loss of Numb expression leads to abnormal activation of Notch signaling pathway, which is closely related to the occurrence and development of colon cancer (3). However, it has been suggested that different subtypes of Numb have different roles in tumors, with NUMB exon 12 (E12) hop isomer p65/p66 promoting epithelial-to-mesenchymal transformation (EMT) and cancer cell migration *in vitro* and promoting cancer metastasis in mice; the p71/p72 isomer acts as a negative regulator of Notch-1 by ubiquitinating the Notch-1 intracellular domain (N1ICD) and promoting its degradation, and the NUMB isoform is considered to be a key regulator of EMT and cancer cell migration (15). In this study, the expression of Notch-1 protein and Numb protein in colon cancer tissues and MLN tissues was detected to analyze the relationship between the two proteins and clinical case factors, so as to provide a possible theoretical basis for finding molecular targets for colon cancer and predicting lymph node metastasis.

Notch signaling pathway promotes the occurrence and development of tumors in most cases but inhibits tumors in some cases. This opposite result may be caused by the different expression levels of Notch-1 protein in different tumor cells and different stages of tumor development (16, 17). This study showed that the expression of Notch-1 protein in colon cancer tissues was significantly higher than that in adjacent tissues (*p* < 0.05), suggesting that the high expression of Notch-1 leads to abnormal activation of Notch signaling pathway, which may be involved in the occurrence and development of colon cancer and play a role in promoting cancer, which is consistent with Xu et al. (18, 19). Many downstream target genes regulated by Notch-1 (such as *CyclinD1*, *Hes1*, *Bcl-2*, *NF-κB*, and *Hey-1*) are closely related to cell proliferation cycle and self-renewal (20, 21). Some studies have found that in mouse intestinal epithelial cells, the lack of Golgi membrane protein 1 causes abnormal activation of Notch, thus affecting the differentiation and maturation of intestinal epithelial cells, leading to the occurrence and development of malignant tumors. Inhibition of Notch abnormal expression by drugs can inhibit the tumor progression of intestinal epithelial cells lacking Golgi membrane protein-1, suggesting that Golgi membrane protein-1 prevents colon tumorigenesis by regulating Notch signaling pathway (22). The Schmidt EM study found that blocking Notch and MAPK signaling pathways by targeted drugs plays a regulatory role in the proliferation and plasticity of different colon cancer cell subsets (23). Pu described the role of newly developed drugs in the regulation of colon cancer by affecting the Notch signaling pathway (24). Therefore, the abnormal activation of Notch may affect the occurrence and development of tumors and also provide a certain theoretical basis for the targeted therapy of colon cancer.

In many cancer types, Numb acts as a tumor suppressor, and its downregulation leads to the development of tumors (3, 25). In this study, it was found that the expression of Numb in para-cancerous tissues was significantly higher than that in cancerous tissues and metastatic lymph nodes (*p*<0.05) and was related to the degree of tissue differentiation, presence or absence of lymph node metastasis, and TNM stage (*p*<0.05), but not related to tumor size and presence or absence of envelope invasion (*p*>0.05). It is suggested that Numb plays a cancer-suppressing role in colon cancer, which is consistent with Zhang et al. (26). Cheng et al. (27) showed that Numb can negatively regulate epithelial–mesenchymal transformation through Wnt signaling pathway, thus inhibiting the development of colorectal cancer, which is consistent with the results of this study. However, the downregulation of Numb expression is negatively correlated with the depth of invasion and tumor size, which is different from the results of this study. It is considered that Numb may be affected by multiple factors or play a role in the development of tumor through multiple signaling pathways. In lung adenocarcinoma, Numb inhibits Notch pathway and epithelial–mesenchymal transformation, inhibiting tumor growth, while in lung squamous cell carcinoma, Numb may promote tumor proliferation (28). Saha et al. (29) found in colon cancer that NUMB may play an important role in the bias effect of Wnt/Notch signaling crosstalk through KRT19. Zhang analyzed the role of Numb in tumor by searching literature with various software, suggesting that in colorectal cancer, NUMBL inhibits Notch pathway in colorectal tumor with unchanged NUMB expression, and the decrease in NUMBL expression leads to increased malignancies and poor prognosis (30). Because Numb has different subtypes and contains different domains, it may play a different role in different tumor tissues and play a cancer suppressor role in colorectal cancer.

Abnormal expression of Notch signaling pathway can affect cell differentiation and induce undifferentiated cells to turn to malignant cells, resulting in the occurrence of tumors, and the degree of differentiation of malignant tumors affects the prognosis of patients (31). In this study, it was found that the positive expression rate of Notch-1 was higher in colon cancer tissues with poor differentiation types (*p* < 0.05), suggesting that the expression of Notch-1 is related to the differentiation types of colon cancer and may be involved in cell differentiation, which is closely related to the occurrence and development of colon cancer.

Lymphatic duct metastasis is the main way of colon cancer recurrence and metastasis and also the key factor of postoperative recurrence of colon cancer (32, 33). The presence of regional lymph node metastasis and the number of metastatic lymph nodes are important determinants of prognosis in patients with colon cancer, so preoperative evaluation of lymph node metastasis is crucial (34). Jepsen used pT1 colorectal cancer with regional lymph node metastasis to investigate the association between the presence of miR-17/92 cluster and LNM, and the results suggested that early regional lymph node metastasis of colon cancer was associated with the high expression level of miR-17/92 cluster members (miR-17-3p, miR-92a) (35). Jiang (36) established a new cell line FDOVL from metastatic lymph nodes of patients with primary platinum-resistant ovarian cancer and found that NOTCH1-pC702fs mutation was only highly expressed in FDOVL cell lines and metastatic lymph nodes, and this mutation promoted the migration and invasion of tumor cells. These effects were significantly inhibited by NOTCH inhibitor LY3039478, suggesting that NOTCH1 mutation may be a driver of lymph node metastasis in ovarian cancer. This study found that the expression of Notch-1 protein in colon cancer tissues with lymph node metastasis was higher than that in colon cancer tissues without lymph node metastasis, suggesting that the overexpression of Notch-1 protein may promote lymph node metastasis of colon cancer. Gonulcu et al. (6) found that the expression status and expression level of Numb and lymph node metastasis and stage are significantly correlated with the survival of colorectal cancer patients. Cox regression analysis showed that lymph node metastasis and downregulation of Numb are independent prognostic factors of colon cancer. In this study, the downregulation of Numb expression is associated with lymph node metastasis in colon cancer tissues, suggesting that the downregulation of Numb expression may promote lymph node metastasis in colon cancer. Yang et al. (37) suggest the following: Numb controls the migration of epithelial cells by regulating intercellular connectivity, and the inhibition of the expression of Numb can promote the migration and invasion of colon cancer cells induced by TGF-β, upregulate the expression of EMT-related molecule Snail, inhibit the expression of E-cadherin, resulting in the destruction of intercellular links, and participate in the invasion and metastasis of colon cancer cells. Ulintz et al. (38) found that both the primary tumor region and the corresponding lymph node metastasis were polyclonal, and the clonal population of each lymph node was different. In some patients, clusters of cancer cells in specific lymph nodes originate from multiple different regions of the tumor. However, there was no significant difference in the expression of Notch-1 protein and Numb protein between colon cancer tissues and MLN tissues (*p*>0.05), suggesting that the malignancy degree of cancer cells did not improve significantly during lymph node metastasis and the biological behavior remained relatively stable. The study also found that poorly differentiated colon cancer patients had a high rate of lymph node metastasis, suggesting that poorly differentiated cancer cells were at high risk of lymph node metastasis. Therefore, in patients with colon cancer, no definite metastatic lymph nodes were found on preoperative imaging, while patients with poor tissue differentiation indicated by preoperative colonoscopy biopsy may be predicted to have a greater potential risk of lymph node metastasis, providing a possible theoretical basis for clinical preoperative staging and follow-up treatment.

The abnormal expression of Notch-1 protein and Numb protein may affect the differentiation degree of tumor cells and lymph node metastasis, thus promoting the occurrence, development, recurrence, and metastasis of colon cancer, providing a possible theoretical basis for exploring the biological behavior of colon cancer cells and targeted therapy. However, the mechanism of Notch-1 and Numb in the lymph node metastasis of colon cancer needs further study.
